# Evaluation of cerebrospinal fluid levels of synaptic vesicle protein, VAMP-2, across the sporadic Alzheimer’s disease continuum

**DOI:** 10.1186/s13195-023-01336-0

**Published:** 2023-10-28

**Authors:** Julie Goossens, Alba Cervantes González, Nele Dewit, Laia Lidón, Juan Fortea, Daniel Alcolea, Alberto Lleó, Olivia Belbin, Eugeen Vanmechelen

**Affiliations:** 1 ADx NeuroSciences NV, Zwijnaarde, Ghent, Belgium; 2 Sant Pau Memory Unit, Neurology Department and IIB-Sant Pau, Hospital de La Santa Creu I Sant Pau, Universitat Autonoma de Barcelona, Barcelona, Spain; 3 Network Center for Biomedical Research in Neurodegenerative Diseases (CIBERNED), Madrid, Spain; 4 Medpace Reference Laboratories (A.A.), Flow Cytometry Unit, Louvain, Belgium

**Keywords:** VAMP-2, SNAP-25, Neurogranin, Alzheimer’s disease, Synapse, Cerebrospinal fluid, Biomarker, Cognitive domains

## Abstract

**Background:**

Synapse loss is an early event that precedes neuronal death and symptom onset and is considered the best neuropathological correlate of cognitive decline in Alzheimer’s disease (AD). Vesicle-associated membrane protein 2 (VAMP-2) has emerged as a promising biomarker of AD-related synapse degeneration in cerebrospinal fluid (CSF). The aim of this study was to explore the CSF profile of VAMP-2 across the AD continuum in relation to core AD biomarkers, other synaptic proteins, neurogranin (Ng) and synaptosomal-associated Protein-25 kDa (SNAP-25) and cognitive performance.

**Methods:**

We developed a digital immunoassay on the Single Molecule Array platform to quantify VAMP-2 in CSF and used existing immunoassays to quantify Ng, SNAP-25 and core CSF AD biomarkers. The clinical study included 62 cognitively unimpaired AD biomarker-negative subjects and 152 participants across the AD continuum from the SPIN cohort (Sant Pau Initiative on Neurodegeneration). Cognitive measures of episodic, semantic, executive and visuospatial domains and global cognition were included. Statistical methods included *χ*^2^ tests, spearman correlation, and ANCOVA analyses.

**Results:**

The VAMP-2 assay had a good analytical performance (repeatability 8.9%, intermediate precision 10.3%). Assay antibodies detected native VAMP-2 protein in human brain homogenates. CSF concentrations of VAMP-2, neurogranin and SNAP-25 were lower in preclinical AD stage 1 compared to controls and higher at later AD stages compared to AD stage 1 and were associated with core AD biomarkers, particularly total tau (adj. *r*^2^ = 0.62 to 0.78, *p* < 0.001). All three synaptic proteins were associated with all cognitive domains in individuals on the AD continuum (adj. *r*^2^ = 0.04 to 0.19, *p* < 0.05).

**Conclusions:**

Our novel digital immunoassay accurately measures VAMP-2 changes in CSF, which reflect AD biomarkers and cognitive performance across multiple domains.

**Supplementary Information:**

The online version contains supplementary material available at 10.1186/s13195-023-01336-0.

## Introduction

Ever since the National Institute on Aging – Alzheimer’s Association (NIA-AA) introduced a new research framework for the definition of Alzheimer’s disease (AD), fluid and imaging biomarkers of hallmark pathologies (Aβ, t-tau, p-tau) have become the main defining biomarkers in AD research. Additional biomarkers, categorized as ‘N’, represent surrogates of underlying neurodegeneration that are not necessarily AD specific, but could have added diagnostic value for disease staging and prognosis [1]. The ‘N’ biomarkers include proxies of cerebral atrophy, axonal degeneration and synaptic dysfunction. Among these, synapse loss is considered to be an early pathological manifestation [2, 3] and the major correlate of cognitive impairment in AD, as evaluated in post-mortem brain tissue of pathologically confirmed cases by electron microscopy [4] and immunohistochemistry targeting the established pre-synaptic protein synaptophysin [3, 5–7].

Objective surrogate biomarkers for AD-related cognitive decline that are easily accessible in fluids are needed; however, very few studies on synaptic proteins have described the spectrum of cognitive impairment in the development of dementia [8]. While the Mini-Mental State Examination (MMSE) is a commonly used cognitive test, the Free and Cued Selective Reminding Test (FCSRT) and the California Verbal Learning Test (CVLT-2) as indicators for episodic memory loss are among the most sensitive in the early stages of AD [9–11].

Synaptic proteins have been evaluated in exploratory CSF cohorts and shown to be altered in subjects with sporadic AD, both by mass spectrometry [12–16] and immunoassays [8, 17–23] but the first steps towards patient monitoring have largely been restricted to CSF neurogranin measurements in clinical trials [24, 25].

A meta-analysis of synaptic pathology on human brain tissue pointed towards a selective molecular vesicular machinery vulnerability [26] and in several AD models, defects in synaptic vesicle recycling were found to be linked to amyloid and tau toxicity in early stages [27, 28]. Consequently, components of the synapse vesicle exocytosis pathway have attracted much attention in the pursuit of synaptic biomarkers for AD. For example, synaptosomal-associated protein-25 kDa (SNAP-25) [8, 18, 20] and synaptotagmin I [29, 30], were increased in CSF even at the earliest preclinical stages [31] and more recently, synaptic vesicle glycoprotein 2A (SV2A) has been proposed as a promising PET imaging target to assess decreased synaptic density in AD patients [32, 33]. In the last few years, vesicle-associated membrane protein-2 (VAMP-2), also known as synaptobrevin-2, has emerged as a promising biofluid marker in AD. VAMP-2 is part of the soluble N-ethylmaleimide-sensitive factor attachment protein receptors (SNARE)-complex, together with SNAP-25 and syntaxin-1, and is the most abundant constituent of pre-synaptic secretory vesicles [34] with a widespread expression throughout the brain [35, 36] and a very specific enrichment in glutamatergic synapses [13, 37]. It also plays a critical role in the post-synaptic trafficking of glutamate receptor subunits, particularly in the CA1 region of the hippocampus [38]. Loss of VAMP-2 in different brain regions has been reported in AD [39, 40] and its presence in CSF has now been documented in multiple, independent studies using mass reaction monitoring (MRM) [13–16, 41]. Moreover, CSF VAMP-2 has been shown to be elevated in CSF from AD patients compared to cognitively unimpaired subjects in multiple clinical cohorts of sporadic AD [13, 15], in a cohort of patients with neuropathologically confirmed AD [41], and in adults with Down syndrome with dementia compared to cognitively unimpaired adults with Down syndrome [42]. Moreover, a head-to-head comparison with eight other synaptic proteins in CSF revealed that VAMP-2 was the best and only synaptic protein to correlate with episodic memory in adults with Down syndrome [42]. On the other hand, CSF VAMP-2 was elevated in AD patients but comparable between patients with neuropathologically confirmed frontotemporal lobar degeneration and cognitively normal individuals, suggesting a certain level of specificity of CSF VAMP-2 for AD [41].

These previous studies of CSF VAMP-2 were performed using our in-house targeted mass spectrometry assay [13, 41, 42]. To facilitate the quantification of VAMP-2 in larger CSF cohorts, we have developed a digital immunoassay for VAMP-2. Here, we provide a characterization of the assay antibodies with different methodologies and describe the analytical characteristics of the assay on the Single Molecule Array (Simoa) platform. Using this assay, we also provide a comprehensive evaluation of CSF VAMP-2 across the sporadic AD continuum in a larger selection (*n* = 214) of the SPIN cohort [43]. We include a full exploration of the association with CSF core AD and other synaptic biomarkers and with measures of cognitive decline.

## Materials and methods

### Study design: Sant Pau Initiative on Neurodegeneration cohort (SPIN)

This is a single-centre, cross-sectional study of CSF levels of the VAMP-2 protein in cognitively unimpaired subjects and participants on the AD continuum selected from the Sant Pau Initiative on Neurodegeneration cohort (SPIN) at Hospital Sant Pau, Barcelona, Spain [43]. The study was approved by the local ethics committee and was conducted in accordance with the Declaration of Helsinki. All participants gave their written informed consent to participate in the study.

### Clinical cohort

All participants from the SPIN cohort [43] were evaluated by neurologists with expertise in neurodegenerative diseases and by neuropsychologists. Specific cognitive tests were administered to assess the main cognitive domains: episodic (Free and Cued Selective Remaining Test), semantic (semantic fluency; 1 min animals), executive function (phonemic fluency; 1 min ‘p’), visuospatial (copy of the figure of the Rey-Osterrieth Complex Figure (ROCF)) using a previously published neuropsychological battery [43], including MMSE and FCSRT. All participants were assessed for core AD biomarkers, namely brain amyloidosis (low CSF levels of Aβ42, CSF Aβ_42:40_ ratio using our local cut-offs or positive amyloid PET imaging), tau pathology and neurodegeneration (high CSF levels of phosphorylated tau and total tau) based on local cut-offs. These cut-offs have high specificity and sensitivity to distinguish AD dementia patients from controls (CSF Aβ42: 916 pg/mL, Aβ42:40 ratio: 0.062, CSF p-tau: 63 pg/mL, CSF t-tau: 456 pg/mL) [44]. Diagnoses of prodromal AD and AD dementia were made according to NIA-AA guidelines [45]. Subjects within the normal range following formal neuropsychological evaluation, when accounting for age and education (mostly recruited among patients’ caregivers), were classified into preclinical AD stages in accordance with NIA-AA guidelines [46]. Inclusion criteria for controls required the absence of a cognitive or neurological disorder (MMSE 27–30, Clinical dementia rating = 0, FCSRT total immediate score > 7, absence of significant impairment in other domains or in daily living activities) and normal CSF AD biomarkers. A subset of samples had VAMP-2 relative quantification using our targeted mass spectrometry assay [13].

### CSF collection and biomarker assessment

CSF samples were collected following international consensus recommendations as previously described [43]. Samples were stored at − 80 °C and were not thawed prior to analysis. Commercially available immunoassays were used on the LUMIPULSE G600II automated platform to determine levels of CSF Aβ_42_, Aβ_40_, total tau (t-tau), and phosphorylated tau 181 (p-tau 181) (Lumipulse® G assays β-Amyloid _1–40_ and _1–42_, t-tau, p-tau 181 from Fujirebio, Ghent, Belgium), while neurogranin was measured with a commercial ELISA (Ng (Neurogranin (Trunc P75) ELISA from EUROIMMUN, Lübeck, Germany)). SNAP-25 CSF levels were determined using an ADx homebrew Simoa assay. Details about the SNAP-25 Simoa assay can be found elsewhere [8], with the remark that detector ADx405 was exchanged for antibody RD042, because of its slightly improved signal-to-noise ratio in Simoa applications. Samples were blinded for clinical diagnosis and randomized before analysis. All samples were measured in batch for the core biomarkers, followed by a separate batch for the synaptic biomarkers.

### VAMP-2 antibody development and characterization

All immunizations, fusion, screening and subcloning protocols were carried out at Biotem (Apprieu, France) according to the ARRIVE (animal research: reporting of in vivo experiments) recommendations [47]. Five OF1 mice were immunized with four low doses (10 μg) of a short keyhole limpet hemocyanin-coupled peptide, S_27_–R_47_, of human VAMP-2 (Uniprot identifier P63027). The spleen was harvested of one selected mouse and its lymphocytes were fused with Sp2/0-Ag14 myeloma cells. Fused cells were plated out in 96-well plates and culture supernatants were screened on biotinylated peptide via ELISA, on HEK293T cell lysate with overexpressed human VAMP-2 (#LY415423, Origene Technologies, Rockville, USA) and on human temporal cortex extracts (Tissue Solutions, Glasgow, UK) via Western blot analysis. One monoclonal antibody (mAb), RD087, was selected and paired with a VAMP-2 specific commercial antibody, D601A, (#13,508, Cell Signaling, Danvers, USA) on Simoa. The epitopes of both antibodies were mapped through indirect ELISA by antibody recognition of short overlapping biotinylated peptides that were coated individually on streptavidin-coated plates. Western blot analysis was performed to analyse reactivity of the mAbs towards recombinant VAMP protein isoforms 1, 2 and 3 (NBC1-18,336, NBC1-18,335, NBC1-18,346, Novus Biologicals, Centennial, USA), and towards native VAMP-2 in soluble human brain homogenate and synaptosome fractions [13]. One hundred nanograms of recombinant VAMP and 50 μL of total soluble brain homogenate or synaptosome were denatured for 10′ at 95 °C, run onto a 10% Tris–glycine gel, blotted and detected with 1 μg/mL of mAb RD087 or D601A and anti-actin (MAB1501R, Merck, Kenilworth, USA), followed by detection with 1:5000 anti-goat or anti-mouse-horseradish peroxidase (Jackson Immuno Research labs, West Grove, USA).

### VAMP-2 immunoassay and partial analytical validation

The VAMP-2 Simoa homebrew assay was developed according to the general Quanterix Homebrew assay guidelines with the homebrew assay development kit components (reference 101,354, Quanterix, Billerica, USA) unless specified otherwise. In brief, 0.25 mg/mL of mouse mAb RD087 was coupled for 2 h at RT to 2E08 paramagnetic beads in 150 μL coupling buffer (50 mM MES, 10 mM NaCl pH 6.2) after activation with 0.05 mg/mL 1-ethyl-3-(3-dimethylaminopropyl) carbodiimide (EDC, #A35391, ThermoFisher Scientific, Waltham, USA). The coated beads were blocked overnight at 4 °C and stored in bead diluent upon further use. Detector mAb D601A was dialyzed overnight into biotinylation buffer (borate pH 8.5) and biotinylated for 1 h at RT with EZ-Link™ Sulfo-NHS-LC-Biotin at 32 × excess (#A39257, Life Technologies, Carlsbad, USA). Next, Tris pH 9 was added for 1 h at a final concentration of 20 mM to stop the reaction. The biotinylated detector was dialyzed back into PBS for further use.

To measure VAMP-2 in CSF, 1E07 active capture beads/mL were combined with 1E07 helper beads/mL in sample diluent (PBS, 0.1% bovine serum albumin, 0.05% Tween-20). Twenty-five microlitres of this bead solution, 100 μL of 1:4 diluted CSF and 20 μL of biotinylated detector (at 1 μg/mL) were incubated for 1 h (80 cadences) in a cuvette, followed by several wash steps. In a second step, 100 μL of 50 pM streptavidin-β-galactosidase was added and incubated for 5 min and 15 s (7 cadences), followed by several wash steps. 50 μL of resorufin β-D-galactopyranoside substrate solution was added to the beads, mixed, and loaded onto the Simoa disc array for imaging. The immunoassay consists of a seven-point peptide calibrator M_1_–A_69_ (500–300–100–50–20–10–5–2 pg/mL) and was characterized for multiple analytical parameters according to previously described guidelines [48]: lower limit of quantification (LLOQ), dilutional linearity, parallelism, spike-recovery, repeatability and intermediate precision. A quality control (QC) panel of three routine CSF samples was measured in four runs on separate days, and at least three times within each run, to analyse precision. The same QC panel was used to qualify the clinical measurements. The LLOQ was calculated based on the mean and variability of sixteen blank measurements. To evaluate parallelism, five CSF samples with high endogenous analyte levels were serially diluted within the calibrator range and measured in duplicate. Dilutional linearity was evaluated using three samples that were spiked with a calibrator concentration above the highest calibration point and diluted three-fold until LLOQ was reached. A zero, low (4.9 pg/mL), medium (22.4 pg/mL) and high (237.8 pg/mL) concentration of calibrator was spiked into five CSF samples with variable endogenous VAMP-2 present and measured in duplicate for spike-recovery assessment. Sample diluent was used as a reference matrix and spiked in the same manner.

### Immunohistochemistry of post-mortem brain tissue

Formalin-fixed paraffin-embedded (FFPE) sections were dewaxed, hydrated and treated with methanol and H_2_O_2_ to inhibit endogenous peroxidase. The sections were boiled in Tris/EDTA buffer pH 9 and blocked with 0.2% bovine serum albumin for 1 h at RT. Then, they were incubated overnight at 4 °C with the primary mAbs RD087 or D601A (#13,508, Cell Signaling, Danvers, USA), diluted 1:200. After washing, sections were incubated 1 h at RT with 1:200 secondary antibody (Goat Anti-Rabbit-HRP, Dako, Glostrup, Denmark). Peroxidase activity was revealed with 3,3’-diaminobenzidine tetrahydrocloride chromogen (liquid DAB + substrate Chromogen System, Dako, Glostrup, Denmark) and stained with haematoxylin (EnVisionTM FLEX haematoxylin, Dako, Glostrup, Denmark). The sections were dehydrated and mounted using DPX (PanReac Applichem, ITW Reagents, Barcelona, Spain).

### Statistical analyses

Statistical analyses were performed in R version 4.0.5. Group differences were compared using *χ*^2^ test for categorical variables, *t*-test or ANCOVA for linear variables. P-values for pairwise age-adjusted means in the ANCOVA were adjusted for multiple testing using the Bonferroni method. Correlation analyses were performed using the Spearman (*ρ*) coefficient. We used receiver operating characteristic curves to determine the diagnostic accuracy via the area under the curve (AUC) and compared ROC curves using the DeLong test. Where regression residuals deviated from a Gaussian distribution (Shapiro–Wilk *p* < 0.05), tests were performed on log2 transformed values, which did not deviate from a Gaussian distribution (Shapiro–Wilk *p* > 0.05).

## Results

### Characterization of VAMP-2 monoclonal antibodies

**Fig. 1:**
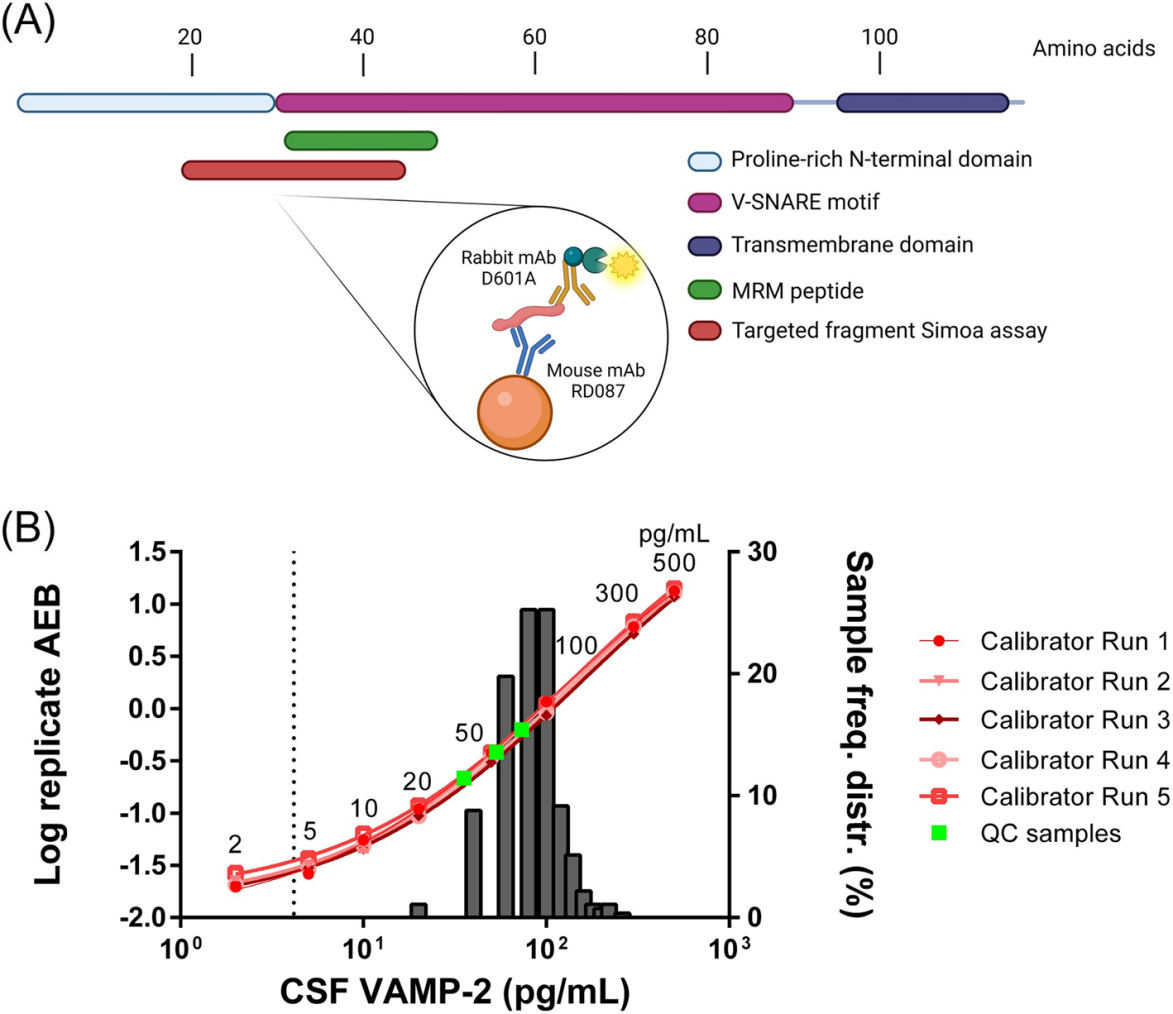
Novel VAMP-2 immunoassay features. **A** Schematic overview of VAMP-2 protein sequence aligned with the protein fragment (red) that is recognized by the VAMP-2 immunoassay consisting of mAbs RD087 and D601A. VAMP-2 protein domains are indicated and assigned with different colours. Highlighted in green is the MRM peptide sequence used by Lleó et al. (2019) [13] to quantify VAMP-2 in CSF of subjects with sporadic Alzheimer’s disease. Created with BioRender.com. **B** Clinical frequency distribution of CSF VAMP-2 levels in patients from the Alzheimer’s continuum (*n* = 152) and cognitively healthy controls (*n* = 62) combined with the assays’ calibration curves (red) from five Simoa runs. The CSF concentrations of three QC samples used in the cohort are highlighted in green and the LLOQ is marked with a dotted line. VAMP-2, vesicle-associated membrane protein-2; mAbs, monoclonal antibodies; MRM, multiple reaction monitoring; LLOQ, lower limit of quantification; QC, quality control

A capture monoclonal antibody RD087 was generated from a mouse immunization with an N-terminal VAMP-2 peptide. This peptide overlaps substantially with the protein sequence of an MRM peptide (human VAMP-2 L_32_–R_47_) used in two independent studies that quantified VAMP-2 changes in AD [13, 15] (Fig. 1A). The antibody clone performed well as a capture antibody in combination with a commercial rabbit monoclonal antibody D601A as detector in the Simoa format resulting in a quantification of all tested CSF samples from clinical and remnant origin (Fig. 1B). Additional antibody characteristics are highlighted in Fig. S1. On Western blot (Fig. S1A), the mAbs detected native full-length VAMP-2 only (one band at approx. 12.6 kDa) in whole human brain homogenates, with an enriched signal in the synaptosomal fraction. D601A had high specificity towards recombinant VAMP-2 while RD087 cross-reacted with recombinant VAMP-1. The detected antigen fragment P_19_–V_43_ is part of the intracellular N-terminal domain and the SNARE motif and the minimal linear epitopes of both mAbs map closely together, with approximately 3 residues apart (Fig. S1B). Both antibodies showed distinct reactivity with the neuropil in grey matter compared to white matter on human cortex tissue slices, which is indicative of synaptic localization (Fig. S1C).

### Analytical and clinical performance of VAMP-2 immunoassay

The VAMP-2 Simoa assay met the acceptance criteria (85–115%) for all studied parameters in the partial analytical validation (Tables S1–S4). It had an LLOQ of 4.2 pg/mL and repeatability and intermediate precision were 8.9% and 10.3%, respectively. The immunoassay was evaluated for cross-reactivity with recombinant VAMP-1 and VAMP-3 homologues but only detected recombinant VAMP-2 (Fig. S2A). Immunoassay protein levels correlated mildly with previous MRM-based quantifications in a subset of the cohort (*ρ* = 0.36, *p* = 0.02) [13] (Fig. S2B). In the clinical evaluation, all VAMP-2 levels could be measured in CSF at dilution factor 4; they were within the quantifiable range and spanned 24–1044 pg/mL (dilution-corrected concentrations) (Fig. 1B). The inter-run coefficient of variation (%CV) of QC samples were 7.5, 8.3 and 8.5%, respectively. The calibrator inter-run %CV and clinical sample intra-run %CV did not exceed 7.6% and 19.2%, respectively.

### Demographics of the clinical cohort

Demographic and clinical data of the 214 participants included in the study are shown in Table 1. The study included cognitively unimpaired subjects and patients in the AD continuum (preclinical (Stage 1 and Stage 2), prodromal AD (pAD) and AD dementia (dAD). The mean age-at-analysis was significantly lower in controls compared to Stage 2 (*p* = 0.03), pAD (*p* < 0.01) and dAD (*p* < 0.01) but not compared to Stage 1 (*p* = 0.2). The male/female ratio was comparable across all groups (*p* = 0.22). The percentage of APOE ε4 carriers was lower (*p* < 0.001) and mean years of education was higher (*p* < 0.001) in controls compared to participants on the AD continuum. As expected, mean MMSE and FCSRT scores were comparable in preclinical Stages 1 and 2 but lower in pAD (*p* < 0.001) and dAD (*p* < 0.001) compared to controls.

**Table 1:** Demographic and clinical data of the participants included in the study

*N*	Controls	AD preclinical stage 1	AD preclinical stage 2	pAD	dAD
	62	30	9	74	39
Age at analysis, years	63 (5.8, 53–74)	61 (6.4, 52–76)	68 (8.3, 57–84)	73 (6, 54–84)	69 (7, 54–85)
% *APOE* ε4 allele carriers	18	33	56	62	59
% female	69	53	44	61	64
CSF Aβ_42:40_ ratio	0.11 (0.01, 0.07–0.12)	0.09 (0.02, 0.04–0.12)	0.04 (0.01, 0.02–0.06)	0.04 (0.01, 0.03–0.06)	0.04 (0.01, 0.03–0.06)
CSF p-tau pg/mL	38 (10, 21–58)	30 (12, 14–56)	119 (80, 67–326)	115 (51, 57–340)	136 (59, 47–384)
CSF t-tau pg/mL	264 (65, 138–438)	204 (79, 85–416)	683 (355, 404–1568)	700 (285, 378–1890)	819 (342, 405–2000)
Education, years	25 (5, 6–20)	15 (3, 8–20)	15 (5, 8–20)	11 (5, 1–20)	11 (4, 3–20)
Global deterioration scale	1 (0.2, 1–2)	1.2 (0.5, 1–3)	1 (0, 1–1)	3 (0, 3–3)	4.2 (0.5, 4–6)
MMSE score	29 (0.9, 26–30)	29 (1, 27–30)	28.4 (1.3, 27–30)	26 (2.2, 19–30)	20.5 (4.7, 9–30)
FCSRT	44.5 (2.9, 35–48)	45.2 (3.6, 32–48)	40.6 (5.9, 33–48)	20.7 (10.8, 0–43)	12 (11, 0–43)
Semantic fluency	20.4 (5.8, 7–33)	18.1 (4.7, 12–27)	21.4 (4.9, 12–33)	9.4 (4.0, 3–16)	12.5 (4.7, 1–22)
Phonemic fluency (executive functioning)	15.8 (4.1, 9–23)	14.6 (6.6, 6–29)	16.0 (4.9, 0–26)	7.5 (4.1, 2–15)	10.5 (4.8, 2–25)
ROCF (visuospatial)	31.6 (2.8, 26–36)	29.2 (5.6, 19–36)	16.3 (4.5, 6–26)	17.6 (13.5, 2–34)	27.6 (6.0, 13–34)

Mean values (standard deviation, range) are given for each variable across clinical and biomarker groups

*AD preclinical stage 1 or 2*, preclinical Alzheimer’s disease stage 1 or 2; *pAD*, prodromal Alzheimer’s disease; *dAD*, AD dementia. *MMSE*, Mini-Mental State Examination; *FCSRT*, Free and Cued Selective Reminding Test. Cut-offs for positivity: CSF Aβ42 < 916 pg/mL, Aβ42:40 ratio < 0.062, CSF p-tau > 63 pg/mL, CSF t-tau: > 456 pg/mL

### Association of synaptic markers with age, sex and APOE ε4 allele

We observed no statistically significant difference in CSF VAMP-2, Ng or SNAP-25 between males and females in the controls (*p* > 0.06). We observed low CSF VAMP-2 (*p* = 0.04) and SNAP-25 (*p* = 0.01) but not Ng (*p* = 0.08) concentrations in females compared to males in the dAD group only. Participants in the pAD group who were carriers of the *APOE* ε4 allele had lower CSF Ng concentrations than non-carriers (*p* = 0.03). This was not the case for VAMP-2 or SNAP-25 or for Ng in another group (*p* > 0.05). CSF VAMP-2 and SNAP-25 were associated with age in controls (adj. *r*^2^ = 0.09, *p* = 0.01, adj. *r*^2^ = 0.05, *p* = 0.04, respectively) and in preclinical Stage 1 (adj. *r*^2^ = 0.32, *p* < 0.001, adj. *r*^2^ = 0.32, *p* < 0.001, respectively). CSF Ng was associated with age in preclinical Stage 1 (adj. *r*^2^ = 0.21, *p* < 0.001) but not in controls (adj. *r*^2^ = 0.03, *p* = 0.08). We observed a significant interaction between age and the *APOE* ε4 allele such that the association of the synaptic markers with age in participants on the AD continuum was weaker in *APOE* ε4 carriers (adj.* r*^2^ = 0.07 to 0.09, *p* < 0.01) compared to noncarriers (adj. *r*^2^ = 0.37 to 0.41, *p* < 0.001).

### CSF synaptic markers have a biphasic profile over the AD continuum compared to controls

**Fig. 2:**
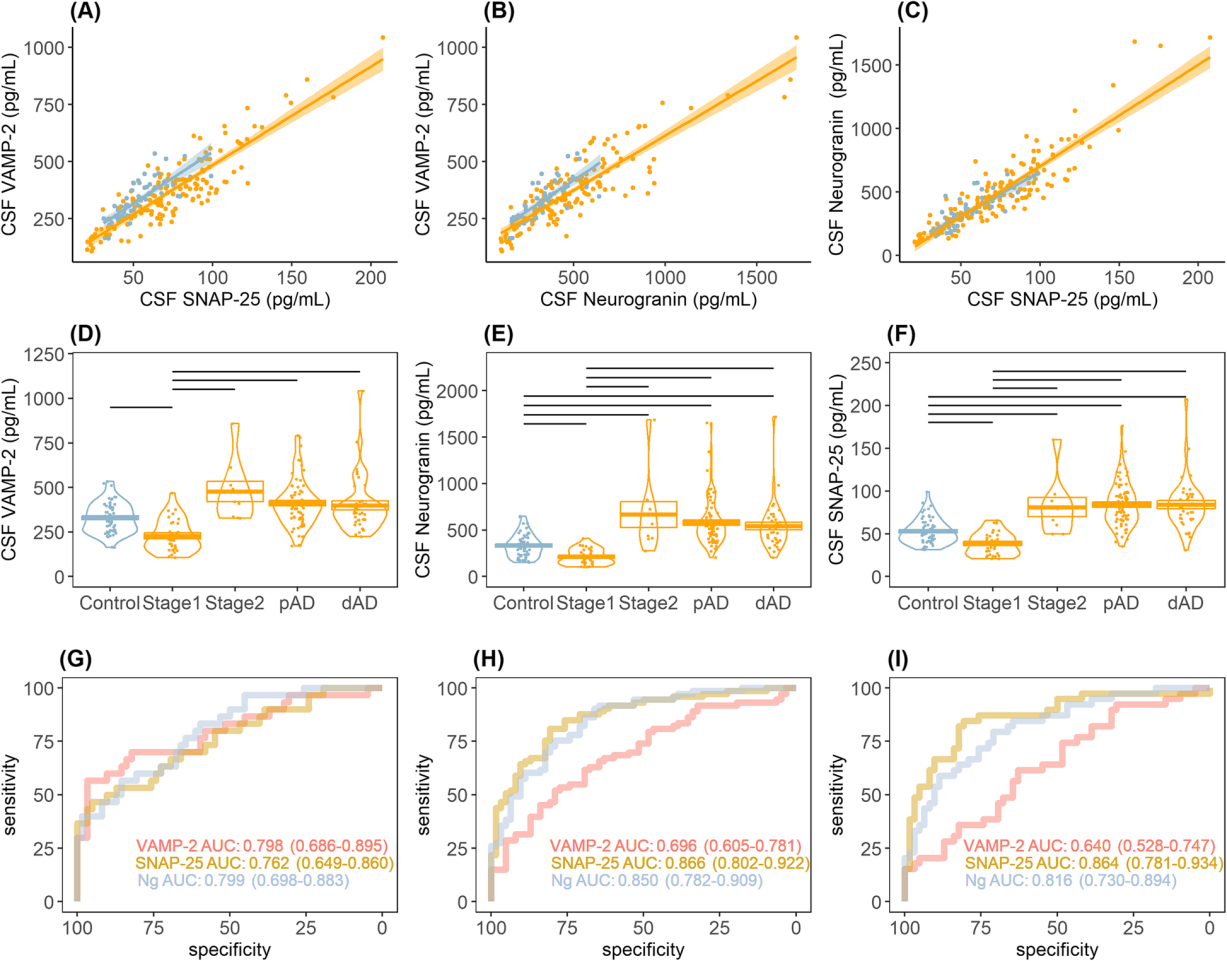
CSF profile of synaptic markers in AD. **A**–**C** Pairwise correlation of VAMP-2, SNAP-25 and neurogranin (Ng) in controls (blue) and in participants on the AD continuum (orange). Linear regression lines are shown for each group. Shaded areas represent standard error of the regression lines. **D**–**F** Violin plots showing mean and standard error (box) CSF levels of VAMP-2, Ng and SNAP-25 in cognitively healthy controls and patients across the AD continuum; (preclinical) Stage 1, (preclinical) Stage 2, prodromal AD (pAD) and AD dementia (dAD). Bars mark comparisons where Bonferonni-adjusted ANCOVA *p* < 0.05 using log2 transformed levels, and adjusted for age. **G**–**I** Receiver operating characteristic (ROC) curves of synaptic proteins at different disease stages: Stage 1 (**G**), pAD (**H**), dAD (**I**)

We observed a strong pair-wise correlation between the three synaptic biomarkers in all groups (*ρ* > 0.88, *p* < 0.001). Figure 2A–C shows the regression lines in participants on the AD continuum (VAMP-2 and SNAP-25 *ρ* = 0.88, *p* < 0.001; VAMP-2 and Ng *ρ* = 0.87, *p* < 0.001; Ng and SNAP-25 *ρ* = 0.89, *p* < 0.001) and controls (VAMP-2 and SNAP-25 *ρ* = 0.79, *p* < 0.001; VAMP-2 and Ng *ρ* = 0.78, *p* < 0.001; Ng and SNAP-25 *ρ* = 0.82, *p* < 0.001). We compared CSF concentrations of the three synaptic biomarkers over the AD continuum. As mean age was elevated across AD stages, we compared CSF VAMP-2, Ng and SNAP-25 across groups including age as a covariate. We observed similar CSF profiles across the AD continuum (Fig. 2D–F). Specifically, CSF levels of VAMP-2 were lower in Stage 1 (*p* < 0.001) but comparable in Stage 2 (adj.*p* = 0.1), pAD (adj.*p* = 0.9) and dAD (adj.*p* = 0.9) compared to controls and elevated in Stage 2 (adj.*p* < 0.01), pAD (adj.*p* < 0.001) and dAD (adj.*p* < 0.001) compared to Stage 1. CSF Ng and SNAP-25 both showed the same biphasic profile when adjusting for age: i.e., higher in Stage 2 (*p* < 0.0001), pAD (*p* < 0.0001) and dAD (*p* < 0.0001) compared to Stage 1 but also lower in Stage 1 (*p* < 0.001) and higher in Stage 2 (adj.*p* < 0.001), pAD (adj.*p* < 0.001) and dAD (adj.*p* < 0.001) compared to controls.

The markers showed diagnostic accuracy to differentiate controls from symptomatic AD (pAD + dAD) as seen in the receiver operating characteristic (ROC) analysis (Fig. 2G–I). SNAP-25 showed high area under the curve (AUC) values to discriminate dAD from controls (AUC = 0.864) and pAD from controls (AUC = 0.866) and performed statistically better than VAMP-2 (AUC = 0.640, 0.696, respectively) in both analyses (*p* < 0.0001). All three proteins showed comparable AUC values to discriminate preclinical Stage 1 from controls (Ng AUC = 0.799, VAMP-2 AUC = 0.798, SNAP-25 AUC = 0.762).

### Association of synaptic biomarkers with core AD biomarkers in CSF

**Fig. 3:**
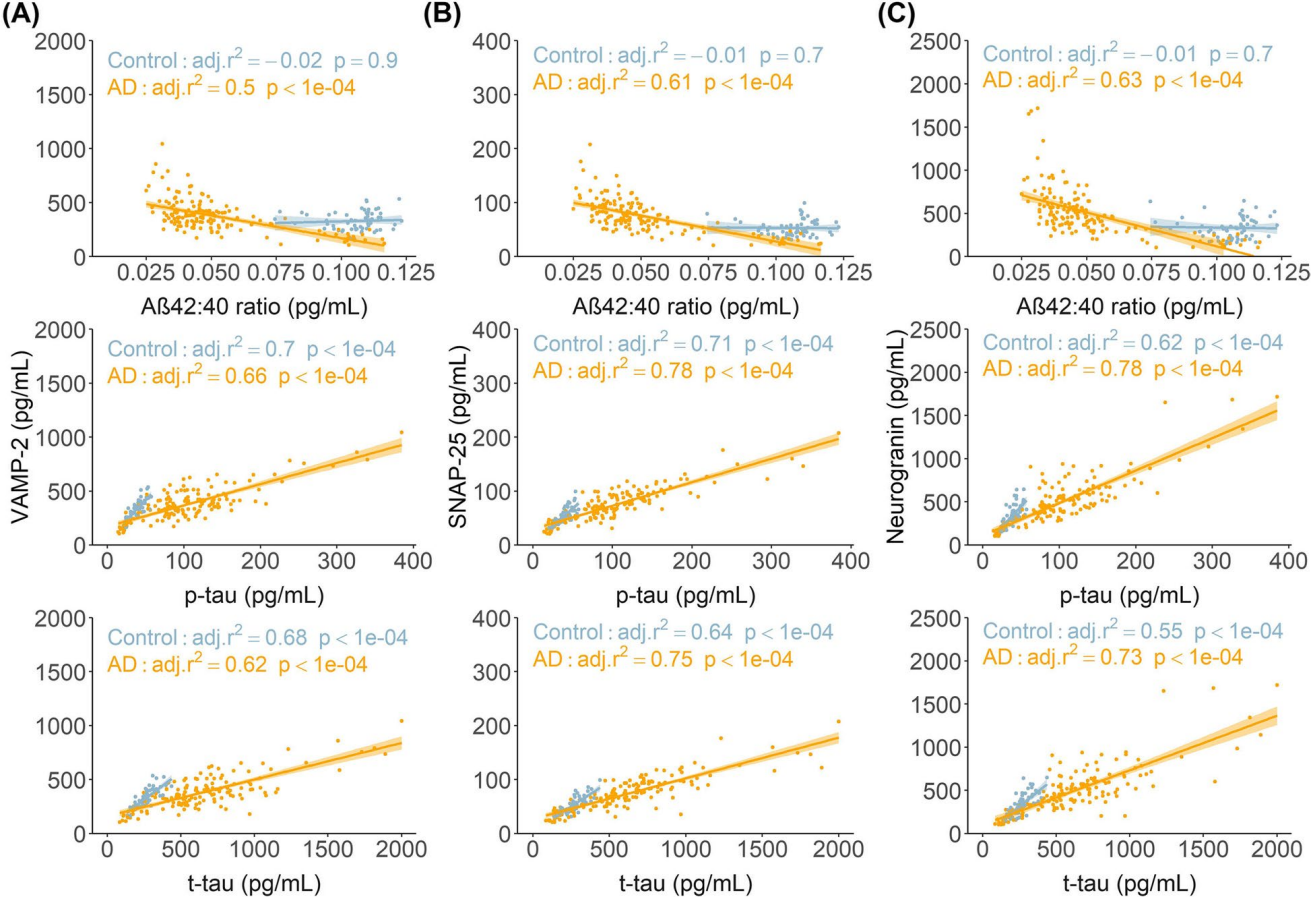
Association of synaptic markers with core AD biomarkers in CSF. CSF VAMP-2 (**A**), SNAP-25 (**B**) and Ng (**C**) are plotted against core CSF AD biomarkers (Aβ_42:40_ ratio, p-tau, total tau) in cognitively healthy controls and AD patients. Linear regression lines are shown for each group. Shaded areas represent standard error of the regression lines

Of the core AD CSF biomarkers, the synaptic proteins were strongly associated with CSF tau markers, p-tau (adj. *r*^2^ = 0.47 to 0.81, *p* < 0.001), and t-tau (adj. *r*^2^ = 0.39 to 0.70, *p* < 0.001) and to a lesser extent with the Aβ_42:40_ ratio (adj. *r*^2^ = 0.11 to 0.49, *p* < 0.001), at all AD stages. Figure 3 plots the regression lines for these associations in all participants on the AD continuum: CSF p-tau (adj. *r*^2^ = 0.66 to 0.78, *p* < 0.001), t-tau (adj. *r*^2^ = 0.62 to 0.75, *p* < 0.001), Aβ_42:40_ ratio (adj. *r*^2^ = 0.5 to 0.63, *p* < 0.001) and in controls, p-tau (adj. *r*^2^ = 0.62 to 0.71, *p* < 0.001), and t-tau (adj. *r*^2^ = 0.55 to 0.68, *p* < 0.001).

### Association of synaptic markers with cognitive performance in AD

**Fig. 4:**
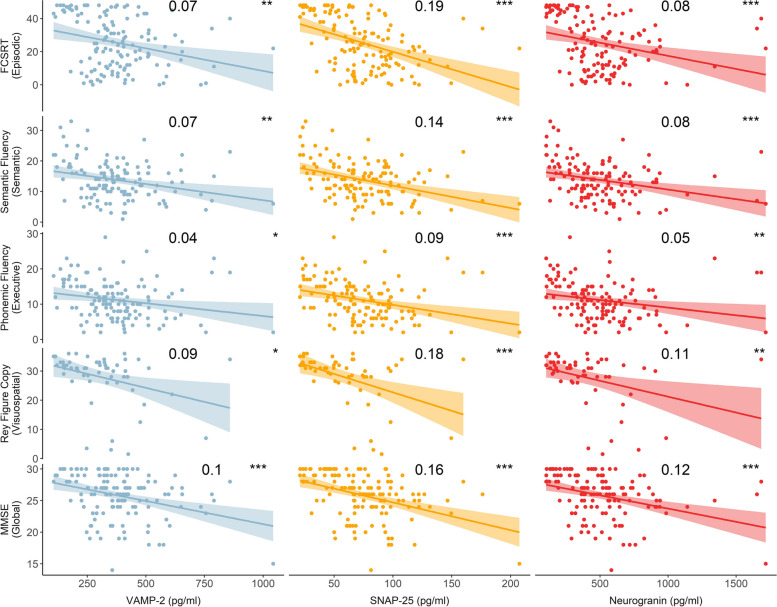
Association of synaptic markers with cognitive performance. CSF VAMP-2, SNAP-25, and Ng are plotted against episodic, semantic, executive, visuospatial domains and global cognition in participants on the Alzheimer’s continuum. Linear regression lines are shown. Shaded areas represent the standard error of the regression lines. FCSRT, Free and Cued Selective Reminding Test, MMSE, mini-mental state examination

We tested for association of the synaptic biomarkers with different memory domains including episodic memory (FCSRT), semantic memory (semantic fluency), executive function (phonemic fluency), visuospatial memory (ROCF test) and global cognition (MMSE) in individuals on the AD continuum, at each AD stage and in controls, adjusting for age and years of education. All three synaptic proteins were associated with all cognitive domains in individuals on the whole AD continuum (Fig. 4) and with executive function in dAD (VAMP-2 adj. *r*^2^ = 0.14, *p* = 0.02, Ng adj. *r*^2^ = 0.12, *p* = 0.03, SNAP-25 adj. *r*^2^ = 0.18, *p* = 0.01). VAMP-2 was associated with episodic memory in controls (adj. *r*^2^ = 0.07, *p* = 0.02). Ng (adj. *r*^2^ = 0.12, *p* = 0.05) and SNAP-25 (adj. *r*^2^ = 0.17, *p* = 0.02) were associated with visuospatial memory in AD stage 1.

## Discussion

We have developed a digital immunoassay to quantify the synaptic protein, VAMP-2, in CSF. Using this assay, we show for the first time that VAMP-2, Ng and SNAP-25 have similar biphasic profiles over the course of the AD continuum compared to controls, showing changes that precede those of other CSF markers of tau-mediated neurodegeneration. The synaptic markers were all individually associated with core AD biomarkers and cognitive decline over multiple domains in participants on the AD continuum. To develop the VAMP-2 assay, we inoculated mice with a short protein fragment of approximately 25 amino acids located in the N-terminal proline-rich domain that showed minimal cross-reactivity towards other VAMP isoforms. Moreover, the sequence of this VAMP-2 fragment largely overlaps with the original MRM peptide that was first detected in CSF. This is because the capture mAb RD087 was explicitly generated against this epitope to facilitate the alignment with ‘gold standard’ mass spectrometry (MS). After all, MS serves as a reference method for the standardization of CSF immunoassays as it helps to develop certified reference materials needed for harmonization, e.g. Aβ_42_ [49]. The new assay had a good analytical performance and it could measure VAMP-2 in all clinical CSF samples in a robust and accurate manner (intermediate precision %CV of approximately 10%).

The availability of immunoassays for other synaptic proteins, Ng and SNAP-25, allowed direct head-to-head comparison of these markers with VAMP-2 in CSF. This is the first time that a biphasic profile of SNAP-25 and Ng has been reported in sporadic AD, but this profile is similar to that of other synaptic proteins (calsyntenin-1, GluR4, neurexin-2A, neurexin-3A, syntaxin-1B and thy-1 cell surface antigen) [13, 41]. In a previous smaller study of VAMP-2 [13] using MRM-based measurements, we observed only a trend towards lower CSF VAMP-2 at this AD stage and elevated levels of CSF VAMP-2 at later AD stages. In this study, higher concentrations of CSF VAMP-2 were associated with higher CSF p-tau181 and t-tau and moderately with lower CSF Aβ_42:40_ ratio throughout the AD continuum. In another cohort where the same VAMP-2 immunoassay was used, controls and AD subjects were divided into four subgroups with increasing p-tau levels. Consistent strong positive associations of VAMP-2 with p-tau were also observed, independent of the disease stage. VAMP-2 was lower in participants with cognitive impairment and positive for amyloid markers but with low p-tau levels (A + ,T-) compared to healthy controls [50]. Combined with the data from this study, we hypothesize that CSF VAMP-2, as well as SNAP-25 and Ng, partially reflect at least two separate mechanisms: (i) synaptic dysfunction with low concentration at normal/low p-tau levels (A + ,T −) which could either be considered a general early-stage process or a process related to a specific biological AD subtype with low p-tau and (ii) tau-mediated neuronal loss which leads to higher concentrations due to release into the CSF. What is causing the decreased CSF levels in A + ,T − subjects without the confounding effect of tau has yet to be determined; it could point towards a reduced structural synaptic density, intracellular sequestration of the protein, a down-regulation of VAMP-2 expression due to altered synaptic activity and/or a decreased synaptic vesicle mobility and release. VAMP-2 and SNAP-25 form part of the SNARE complex, and VAMP-2 is the most abundant constituent of synaptic secretory vesicles [35] with a widespread expression throughout the brain [36, 37]. Perturbation of SNARE complex assembly has also been reported in post-mortem brains of AD patients [51] which may be caused by directly interfering amyloid-beta oligomers that impair SNARE-mediated exocytosis [52, 53].

Individually, the three synaptic markers were mildly associated with measures of cognitive impairment across all domains studied (episodic, semantic, executive, visuospatial and global cognition) in individuals on the AD continuum.

In a previous study comparing the expression of seven presynaptic proteins in postmortem tissue, VAMP-2 was the only protein decreased in all tested brain regions (CA1 region in the hippocampus, the occipital cortex, the entorhinal cortex and caudate nucleus) of AD compared to non-neurodegenerative controls and low VAMP-2 expression was associated with MMSE and FCSRT scores in the hippocampus and entorhinal cortex [40]. In the same study, SNAP-25 was comparable between AD and controls in all brain regions tested. Taken together, future evaluation of the associations of CSF VAMP-2 and SNAP-25 with in vivo neural correlates (e.g. hippocampal atrophy) of declining episodic memory and other cognitive functions in AD patients as measured with structural MRI [54], could provide valuable information.

Emerging blood biomarkers are more easily accessible for widespread clinical use than CSF biomarkers and an extensive unbiased proteomics study has shown that synaptic proteins including Ng and SNAP-25 are present in plasma [55]. Unfortunately, measurements of Ng in blood do not have diagnostic value for AD as levels are high and unrelated to altered CSF neurogranin, probably due to the contribution of peripherally expressed neurogranin peptides [17]. Also, current SNAP-25 immunoassay formats lack sensitivity to pick up SNAP-25 fragments in plasma [20] so it is worthwhile exploring VAMP-2 as a potential marker for synaptic integrity in blood in the future.

A limitation of this study is the cross-sectional design, particularly in the analysis of cognitive decline. Thus longitudinal studies, measuring several cognitive scores on multiple time points, preferably combined with an additional CSF samples will help to fully establish the prognostic value of VAMP-2 concentrations in the AD continuum. Another limitation is the relatively small sample size for the preclinical AD stages which show decreased synaptic protein levels in A + T − individuals in this study. To validate these findings larger studies are needed with well-defined criteria for preclinical AD.

In conclusion, we have developed a novel digital immunoassay with good specificity and sensitivity to measure CSF VAMP-2 levels in subjects with sporadic AD. CSF VAMP-2 performed similarly to CSF SNAP-25 and Ng, showing changes early in the disease process that correlate with core AD biomarkers and multiple cognitive domains.

### Supplementary Information

**Additional file 1: Figure S1.** Characterization of monoclonal antibodies used in immunoassay. (**A**) Western blot showing reactivity of mAb D601A (left panel) and RD087 (middle panel) towards recombinant VAMP protein isoforms and native VAMP. VAMP-1, -2 and -3 (lanes 1, 2 and 3 resp.) were loaded on a gel besides whole homogenates (lanes 4 and 6) and a synapse enriched fraction (lane 5) of a post-mortem human cortex.  The homogenates are also shown on SDS-PAGE (right panel). White arrowhead: actin, black arrowhead: VAMP. (**B**) Peptide scan to map minimal epitopes of D601A (red) and RD087 (green). Either antibody was added to individually coated biotinylated peptides with sequential overlap in indirect ELISA (pt435-pt447, upper panel). (**C**) Immunohistochemistry on human frontal cortex using RD087 (upper panels) or D601A (lower panels). Staining with either mAb locates predominantly to the neuropil in grey matter only, which is consistent with synaptic localization. VAMP, vesicle-associated membrane protein; mAb, monoclonal antibody.

**Additional file 2: Figure S2.** Specificity analysis of VAMP-2 Simoa assay. (**A**)Recombinant VAMP-1, VAMP-2, VAMP-3 were serially diluted in sample diluent and measured in parallel with the VAMP-2 homebrew assay.  (**B**) Correlation of VAMP-2 CSF concentrations obtained with Simoa versus MRM [13] on a subset (n=41) of the SPIN cohort. VAMP, vesicle-associated membrane protein; MRM, multiple reaction monitoring; ρ, Spearman rank correlation coefficient; CI, confidence interval.

**Additional file 3: Table S1.** Repeatability and intermediate precision of VAMP-2 assay on three routine CSF samples (QC1-QC3). Sr = repeatability standard deviation, SRW = intermediate precision standard deviation. **Table S2.** Spike-recovery of VAMP-2 assay on four routine CSF samples spiked with low, medium and high concentration of calibrator peptide. **Table S3.** Dilutional linearity using three routine CSF samples spiked with a concentration above the highest calibration point and diluted three-fold into measuring range. Mean percent linearity is calculated as 100 * (observed concentration at dilution X) * (dilution factor X) / (observed concentration at dilution X-1) * (dilution factor (X-1)). DF, dilution factor. **Table S4.** Parallelism of five CSF samples serially diluted in duplicate within the quantifiable range of the calibration curve. DF, dilution factor.

## Data Availability

The dataset(s) supporting the conclusions of this article is(are) included within the article (and its additional file(s).

## Abbreviations

%CV: Coefficient of variation (percentage)
Aβ_42:40_: Amyloid β 42 over amyloid β 40 ratio
AD: Alzheimer’s disease
AEB: Average number of enzymes per bead
AIC: Akaike information criteria
APOE ε4: Apoliprotein E ε4 allele
AUC: Area under the curve
CA1: Cornu ammonis 1
CSF: Cerebrospinal fluid
CVLT-2: California Verbal Learning Test-2
DAB: 3,3′-Diaminobenzidine
dAD: AD dementia
EDC: 1-Ethyl-3-(3-dimethylaminopropyl)carbodiimide
FFPE: Formalin-fixed paraffin-embedded
FCSRT: Free and Cued Selective Reminding Test
LLOQ: Lower limit of quantification
mAb: Monoclonal antibody
MMSE: Mini-Mental State Examination
MRM: Mass reaction monitoring
MS: Mass spectrometry
Ng: Neurogranin
NIA-AA: National Institute on Aging – Alzheimer’s Association
pAD: Prodromal AD
QC: Quality control
ROCF: Rey–Osterrieth complex figure
ROC: Receiver operating characteristic
RT: Room temperature
SNAP-25: Synaptosomal-associated protein-25 kDa
SNARE: Soluble N-ethylmaleimide-sensitive factor attachment protein receptors
SPIN: Sant Pau Initiative on Neurodegeneration
SV2A: Synaptic vesicle glycoprotein 2A
t-tau: Total tau
p-tau: Phosphorylated tau
VAMP-2: Vesicle-associated membrane protein 2
