# Supplementary figures and images for: Evaluation of cerebrospinal fluid levels of synaptic vesicle protein, VAMP-2, across the sporadic Alzheimer’s disease continuum
Source: Alzheimers Res Ther. 2023 Oct 28;15:186. doi: 10.1186/s13195-023-01336-0 (PMC10612328; doi:10.1186/s13195-023-01336-0)

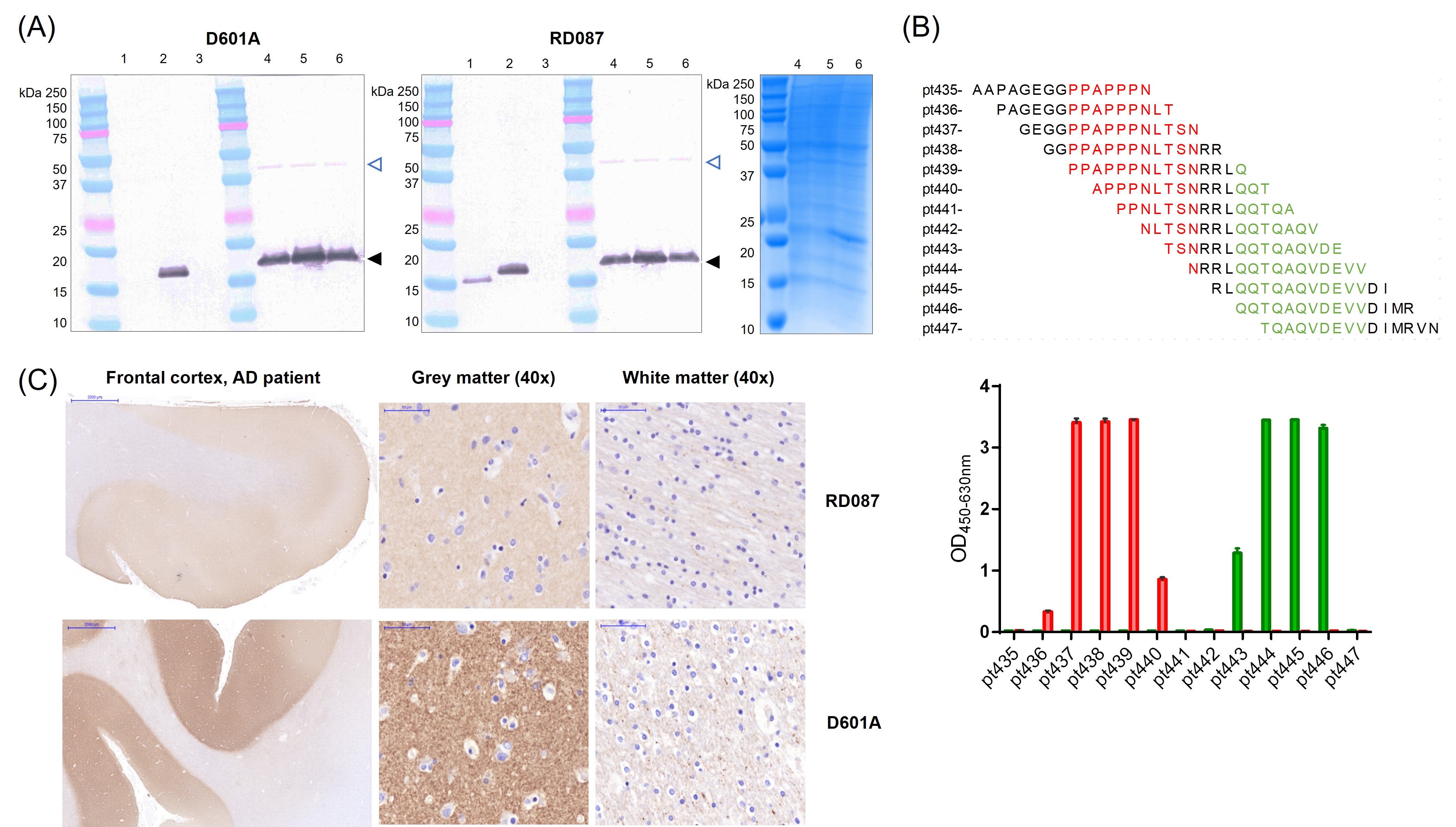

Supplement: Supplementary file 1 — Additional file 1: Figure S1. Characterization of monoclonal antibodies used in immunoassay. (A) Western blot showing reactivity of mAb D601A (left panel) and RD087 (middle panel) towards recombinant VAMP protein isoforms and native VAMP. VAMP-1, -2 and -3 (lanes 1, 2 and 3 resp.) were loaded on a gel besides whole homogenates (lanes 4 and 6) and a synapse enriched fraction (lane 5) of a post-mortem human cortex. The homogenates are also shown on SDS-PAGE (right panel). White arrowhead: actin, black arrowhead: VAMP. (B) Peptide scan to map minimal epitopes of D601A (red) and RD087 (green). Either antibody was added to individually coated biotinylated peptides with sequential overlap in indirect ELISA (pt435-pt447, upper panel). (C) Immunohistochemistry on human frontal cortex using RD087 (upper panels) or D601A (lower panels). Staining with either mAb locates predominantly to the neuropil in grey matter only, which is consistent with synaptic localization. VAMP, vesicle-associated membrane protein; mAb, monoclonal antibody. [file 13195_2023_1336_MOESM1_ESM.jpg]

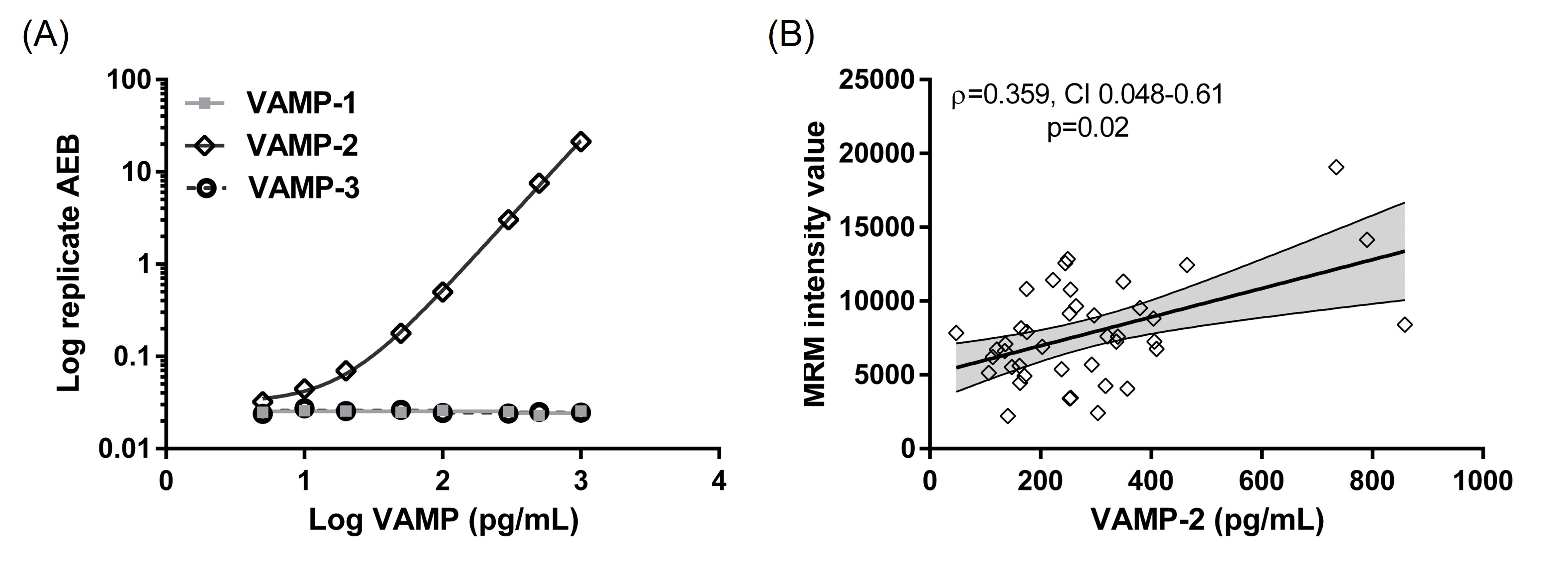

Supplement: Supplementary file 2 — Additional file 2: Figure S2. Specificity analysis of VAMP-2 Simoa assay. (A)Recombinant VAMP-1, VAMP-2, VAMP-3 were serially diluted in sample diluent and measured in parallel with the VAMP-2 homebrew assay. (B) Correlation of VAMP-2 CSF concentrations obtained with Simoa versus MRM [13] on a subset (n=41) of the SPIN cohort. VAMP, vesicle-associated membrane protein; MRM, multiple reaction monitoring; ρ, Spearman rank correlation coefficient; CI, confidence interval. [file 13195_2023_1336_MOESM2_ESM.jpg]
