# Supplementary material for: Evaluation of cerebrospinal fluid levels of synaptic vesicle protein, VAMP-2, across the sporadic Alzheimer’s disease continuum
Source: Alzheimers Res Ther. 2023 Oct 28;15:186. doi: 10.1186/s13195-023-01336-0 (PMC10612328; doi:10.1186/s13195-023-01336-0)
Supplement: Supplementary file 3 — Additional file 3: Table S1. Repeatability and intermediate precision of VAMP-2 assay on three routine CSF samples (QC1-QC3). Sr = repeatability standard deviation, SRW = intermediate precision standard deviation. Table S2. Spike-recovery of VAMP-2 assay on four routine CSF samples spiked with low, medium and high concentration of calibrator peptide. Table S3. Dilutional linearity using three routine CSF samples spiked with a concentration above the highest calibration point and diluted three-fold into measuring range. Mean percent linearity is calculated as 100 * (observed concentration at dilution X) * (dilution factor X) / (observed concentration at dilution X-1) * (dilution factor (X-1)). DF, dilution factor. Table S4. Parallelism of five CSF samples serially diluted in duplicate within the quantifiable range of the calibration curve. DF, dilution factor. [file 13195_2023_1336_MOESM3_ESM.docx]

**Table S1**.

| **Reference sample** | **[VAMP-2] (pg/mL)** | **S_r_ (pg/mL)** | **Repeatability (%CV_r_)** | **S_RW_ (pg/mL)** | **Intermediate precision (%CV_RW_)** |
| --- | --- | --- | --- | --- | --- |
| QC1 | 11,3 | 1,0 | 9,2 | 1,1 | 9,5 |
| QC2 | 15,9 | 1,0 | 6,4 | 1,4 | 8,8 |
| QC3 | 38,9 | 4,3 | 11,1 | 4,9 | 12,6 |
| Mean |  |  | 8,9 |  | 10,3 |

Repeatability and intermediate precision of VAMP-2 assay on three routine CSF samples (QC1-QC3). Sr = repeatability standard deviation, SRW = intermediate precision standard deviation

**Table S2.**

| **Recovery percentage (%)** | **Low spike (4,9 pg/mL)** | **Medium spike (22,4 pg/mL)** | **High spike (237,8 pg/mL)** |
| --- | --- | --- | --- |
| CSF1 | 124,7 | 107,1 | 100,8 |
| CSF2 | 118,2 | 95,0 | 103,4 |
| CSF3 | 129,1 | 115,4 | 95,2 |
| CSF4 | 124,9 | 120,1 | 104,3 |
| Mean recovery percentage | 124,2 | 109,4 | 100,9 |

Spike-recovery of VAMP-2 assay on four routine CSF samples spiked with low, medium and high concentration of calibrator peptide.

**Table S3.**

| **Dilution factor** | **CSF spike 1** | | | | **CSF spike 2** | | | | **CSF spike 3** | | | |
| --- | --- | --- | --- | --- | --- | --- | --- | --- | --- | --- | --- | --- |
|  | **[VAMP-2] (pg/mL)** | **[VAMP-2]*DF (pg/mL)** | **%Linearity** | **%CV** | **[VAMP-2] (pg/mL)** | **[VAMP-2]*DF (pg/mL)** | **%Linearity** | **%CV** | **[VAMP-2] (pg/mL)** | **[VAMP-2]*DF (pg/mL)** | **%Linearity** | **%CV** |
| 1 | 625,2 | 625,2 | - | 4 | 628,5 | 628,5 | - | 4 | 641,3 | 641,3 | - | 5 |
| 3 | 277,5 | 832,6 | 133,2 | 3 | 292,4 | 877,1 | 139,6 | 3 | 291,3 | 873,8 | 136,3 | 2 |
| 9 | 100,0 | 900,3 | 108,1 | 1 | 92,4 | 832,0 | 94,9 | 2 | 85,8 | 772,2 | 88,4 | 9 |
| 27 | 37,0 | 999,5 | 111,0 | 8 | 28,1 | 758,6 | 91,2 | 25 | 31,2 | 841,8 | 109,0 | 3 |
| 81 | 12,0 | 969,5 | 97,0 | 12 | 11,9 | 962,7 | 126,9 | - | 10,1 | 819,8 | 97,4 | 3 |
| 243 | 4,4 | 1063,5 | 109,7 | 24 | 3,5 | 850,6 | 88,4 | 9 | 3,3 | 802,1 | 97,8 | 0 |
|  |  |  |  |  |  |  |  |  |  |  |  |  |

Dilutional linearity using three routine CSF samples spiked with a concentration above the highest calibration point and diluted three-fold into measuring range. Mean percent linearity is calculated as 100 * (observed concentration at dilution X) * (dilution factor X) / (observed concentration at dilution X-1) * (dilution factor (X-1)). DF, dilution factor.

**Table S4.**

| **Sample** | **DF** | **Mean [VAMP-2] (pg/mL)** | **SD** | **%CV** | **[VAMP-2] * DF (pg/mL)** | **% Recovery** |
| --- | --- | --- | --- | --- | --- | --- |
| CSF 1 | 4 | 48,8 | 1,8 | 4 | 195,3 | - |
|  | 6 | 32,9 | 0,4 | 1 | 197,3 | 101,0 |
|  | 8 | 26,3 | 1,2 | 5 | 210,0 | 107,5 |
|  | 10 | 19,6 | 0,4 | 2 | 195,8 | 100,2 |
|  | 12 | 16,5 | 0,2 | 2 | 198,4 | 101,6 |
|  | 15 | 12,2 | 1,3 | 11 | 182,6 | 93,5 |
| CSF 2 | 4 | 53,0 | 1,5 | 3 | 211,9 | - |
|  | 6 | 35,9 | - | - | 215,7 | 101,8 |
|  | 8 | 27,8 | 0,5 | 2 | 222,7 | 105,1 |
|  | 10 | 20,2 | 0,7 | 3 | 202,3 | 95,5 |
|  | 12 | 18,3 | 0,1 | 0 | 219,0 | 103,3 |
|  | 15 | 13,5 | 1,0 | 7 | 203,2 | 95,9 |
| CSF 3 | 4 | 57,0 | 1,3 | 2 | 228,1 | - |
|  | 6 | 42,2 | 3,2 | 8 | 253,2 | 111,0 |
|  | 8 | 33,0 | - | - | 264,3 | 115,9 |
|  | 10 | 24,2 | 2,4 | 10 | 241,6 | 105,9 |
|  | 12 | 22,3 | 0,8 | 3 | 267,9 | 117,5 |
|  | 15 | 15,7 | 1,9 | 12 | 235,0 | 103,0 |
| CSF 4 | 4 | 50,0 | 0,4 | 1 | 200,1 | - |
|  | 6 | 34,3 | 0,3 | 1 | 205,9 | 102,9 |
|  | 8 | 26,0 | 1,0 | 4 | 207,7 | 103,8 |
|  | 10 | 18,7 | 0,5 | 3 | 187,4 | 93,7 |
|  | 12 | 15,9 | 1,2 | 7 | 191,3 | 95,6 |
|  | 15 | 12,4 | 1,1 | 9 | 186,1 | 93,0 |
| CSF 5 | 4 | 61,8 | 6,5 | 11 | 247,0 | - |
|  | 6 | 46,7 | 0,0 | 0 | 280,0 | 113,4 |
|  | 8 | 27,6 | - | - | 220,7 | 89,4 |
|  | 10 | 29,4 | 1,0 | 4 | 294,1 | 119,1 |
|  | 12 | 24,5 | 1,6 | 6 | 294,1 | 119,1 |
|  | 15 | 19,1 | 1,6 | 9 | 286,4 | 115,9 |

Parallelism of five CSF samples serially diluted in duplicate within the quantifiable range of the calibration curve. DF, dilution factor
